# Ocular Manifestations of Celiac Disease: Current Evidence and Clinical Implications

**DOI:** 10.3390/nu17233781

**Published:** 2025-12-02

**Authors:** Monika Senterkiewicz, Anna Szaflarska-Popławska, Bartłomiej J. Kałużny

**Affiliations:** 1 Department of Ophthalmology, Collegium Medicum, Nicolaus Copernicus University in Torun, ul. Ujejskiego 75, 85-168 Bydgoszcz, Poland; 2 Department of Pediatric Endoscopy and Gastrointestinal Function Testing, Collegium Medicum in Bydgoszcz, Nicolaus Copernicus University in Torun, 85-094 Bydgoszcz, Poland; aszaflarska@wp.pl; 3 Department of Ophthalmology, Collegium Medicum, Nicolaus Copernicus University, ul. M. Skłodowskiej-Curie 9, 85-094 Bydgoszcz, Poland

**Keywords:** celiac disease, ocular manifestation, eye diseases, immune dysregulation, nutritional deficiencies, gluten-free diet, ophthalmic disorders, review

## Abstract

**Background:** Celiac disease (CD) is a systemic autoimmune disorder triggered by gluten exposure in genetically predisposed individuals. Beyond gastrointestinal symptoms, CD is increasingly recognized to affect extraintestinal organs, including the eye. **Methods:** A PubMed, Cochrane, Web of Science, and Scopus databases search up to April 2025 was conducted to identify studies on ocular involvement in CD. **Results:** Large population-based cohorts have demonstrated an increased risk of cataract and uveitis in individuals with CD. Cross-sectional and case–control studies further report reduced tear break-up time and decreased Schirmer test values, indicating tear film instability and associated ocular surface abnormalities. Additional findings include reduced anterior chamber depth and volume, alterations in subfoveal and peripapillary choroidal thickness, thinning of the retinal nerve fiber layer, and microvascular changes such as reduced superficial and deep capillary plexus densities. Furthermore, deficiencies of vitamins A, D, B12, and iron have been consistently associated with structural and functional ocular alterations, underscoring the contribution of impaired nutrient absorption. **Conclusions:** Ocular involvement in CD likely reflects the interplay of immune dysregulation, nutritional deficiencies, and microvascular alterations. Ophthalmic referrals should be considered in CD patients presenting with ocular symptoms. Early recognition and regular monitoring may facilitate timely diagnosis, improve visual outcomes, and support normal ocular development.

## 1. Introduction

Celiac disease (CD) is a systemic autoimmune disorder that can occur after exposure to gluten and related prolamins in genetically predisposed individuals. It is characterized by a variety of clinical symptoms, the presence of specific serum antibodies, HLA-DQ2 and/or HLA-DQ8 haplotypes, and enteropathy. Exposure to gluten peptides activates both innate and adaptive immunity, particularly Th1 and Th17 pathways, resulting in the release of pro-inflammatory cytokines (e.g., TNF-α, IL-6, IL-15, IL-17) and the generation of disease-specific autoantibodies such as anti-gliadin and anti-transglutaminase antibodies [1]. The current prevalence of positive serological tests specific to CD is 1.4%, while histopathologically confirmed CD stands at 0.7% worldwide [2]. The clinical symptoms of CD are diverse and can affect both the gastrointestinal tract and other organs [3]. For this lifelong disease, the only effective treatment is strict adherence to a gluten-free diet (GFD), which involves eliminating products containing barley, wheat, and rye. In most patients, this leads to clinical improvement, normalization of blood antibody levels, and regeneration of the small intestinal mucosa [4]. However, a gluten-free diet may result in deficiencies of both macro- and micronutrients, such as vitamin D, B12, E, iron, folic acid, magnesium, potassium, sodium, and dietary fiber. At the same time, the gluten-free diet tends to be higher in saturated fatty acids and carbohydrates. Therefore, patients with CD require ongoing monitoring to assess nutritional status, the presence of disease symptoms, and compliance with the gluten-free regimen [5,6,7].

CD is associated with a generalized increase in mortality risk (odds ratio (OR) 1.21; 95% confidence interval (CI): 1.17–1.25) and increases the probability of death due to oncological, cardiovascular, and respiratory diseases [8]. Among the complications of untreated or improperly treated CD are ulcerative jejunoileitis, enteropathy associated with non-Hodgkin T-cell lymphoma (EATL), small intestine adenocarcinoma, and hyposplenism. This disorder increases the risk of developing autoimmune diseases and infections by encapsulated bacteria such as *Haemophilus influenzae*, *Pneumococcus*, and *Meningococcus* [9].

According to the current nomenclature, five forms of CD are distinguished: classical, non-classical, subclinical, asymptomatic, and potential [10]. An overview of these clinical subtypes with definitions and clinical presentations is provided in Table 1.

Over the past years, there has been an increase in the occurrence of extraintestinal symptoms, affecting up to 60% of children with CD. These include short stature, delayed puberty, anemia, osteoporosis, liver dysfunction, headaches, epilepsy, aphthous stomatitis, enamel hypoplasia, dermatitis herpetiformis, psychiatric disorders, and many others [11]. Moreover, the clinical profile of CD has shifted over time, showing a higher prevalence of non-classical and subclinical phenotypes [12,13].

Recently, an expanding body of evidence has linked CD with various ophthalmic disorders. Abnormalities have been described in both the anterior and posterior segments of the eye [14,15]. Several investigations have employed optical coherence tomography (OCT), a non-invasive imaging technique that provides high-resolution, cross-sectional views of the retina, choroid, and optic nerve. This imaging technique can detect retinal microvascular abnormalities, as well as structural alterations involving the neuroretinal and choroidal layers [16,17]. Collectively, these findings underscore OCT and OCT angiography (OCTA) as sensitive, non-invasive tools with significant potential to enhance early diagnosis and longitudinal monitoring of autoimmune diseases affecting the eye, including CD [18,19,20].

Available studies indicate that several mechanisms may contribute to ophthalmic manifestations in CD, including impaired nutrient absorption, immune-mediated processes, and vascular alterations within ocular tissues [14,15]. Malabsorption of vitamins A, D, B12, folate, and iron is well documented in untreated CD and may persist in some individuals despite adherence to a gluten-free diet [5,6]. These micronutrients are essential for maintaining tear film stability, epithelial integrity, retinal metabolism, and microvascular function [21,22,23,24,25]. Thus, ocular changes in CD may result not only from immune dysregulation but also from nutritional deficiencies, highlighting the importance of considering both pathways when assessing potential eye involvement [14,15].

Despite growing interest, current evidence remains fragmented across small observational cohorts, case reports, and only a few large population-based studies. This dispersion limits clinical recognition and hinders the development of standardized recommendations for screening or management. Considering that ocular abnormalities may occasionally precede gastrointestinal symptoms or represent the first detectable sign of CD, a comprehensive synthesis of existing data is needed to clarify their clinical relevance and guide future research.

The aim of this review was to synthesize current evidence on ocular manifestations of celiac disease, with a focus on structural and functional alterations, underlying mechanisms, and their potential clinical implications.

## 2. Materials and Methods

### 2.1. Database Sources and Search Strategy

A comprehensive literature search was conducted in PubMed, Cochrane, Web of Science, and Scopus databases up to April 2025. The following search strategy was applied: (“celiac disease” OR “coeliac disease”) AND (“ophthalmic” OR “ocular” OR “eye” OR “uveitis” OR “choroid” OR “retina” OR “optical coherence tomography”). The search was limited to English-language publications. Non-relevant articles, duplicates, and records without abstracts or full text were excluded during manual screening. Study selection was performed independently by two reviewers. Titles and abstracts were screened for eligibility, and full texts were retrieved for potentially relevant records. Discrepancies in study inclusion were resolved through discussion and consensus.

### 2.2. Eligibility Criteria

Eligibility criteria were defined using the PICO framework. The population of interest included pediatric and adult patients with confirmed CD. The exposure was the diagnosis of CD, while the comparisons were healthy controls or CD patients stratified by clinical features such as disease duration or diet adherence. The outcomes of interest were ophthalmic findings assessed by clinical examination or imaging modalities, including confocal microscopy, OCT, and OCTA.

Studies were included if they involved human participants and reported ophthalmic outcomes in patients with confirmed CD. Exclusion criteria were:

(a) studies conducted in experimental animals or in vitro;
(b) publications not reporting ophthalmic outcomes;
(c) studies without a confirmed CD diagnosis or with insufficient diagnostic criteria;
(d) duplicate datasets;
(e) non-original publications (editorials, commentaries, conference abstracts without full data);
(f) non-English full texts or studies where the full text was not available.

Both observational studies and case reports were eligible for inclusion.

### 2.3. Study Outcomes

The primary outcomes were structural and functional ocular alterations in patients with CD, assessed by clinical examination or imaging modalities.

### 2.4. Critical Appraisal

Given the narrative design of this review, no formal risk-of-bias scoring was performed. Instead, all included studies were critically appraised with particular emphasis on study design (population-based cohort, cross-sectional, case–control, case series, case report); sample size and adequacy of control groups; methodology and reproducibility of ophthalmic assessments; adjustment for potential confounders (e.g., age, duration of the disease, adherence to gluten-free diet). Population-based cohort studies were classified as high-level evidence. Cross-sectional studies were considered moderate-level evidence, whereas case reports or case series were regarded as low-level evidence and interpreted with caution. This stratification enabled a more balanced synthesis and appropriate weighting of the available findings.

## 3. Results

### 3.1. Literature Search

**Figure 1:**
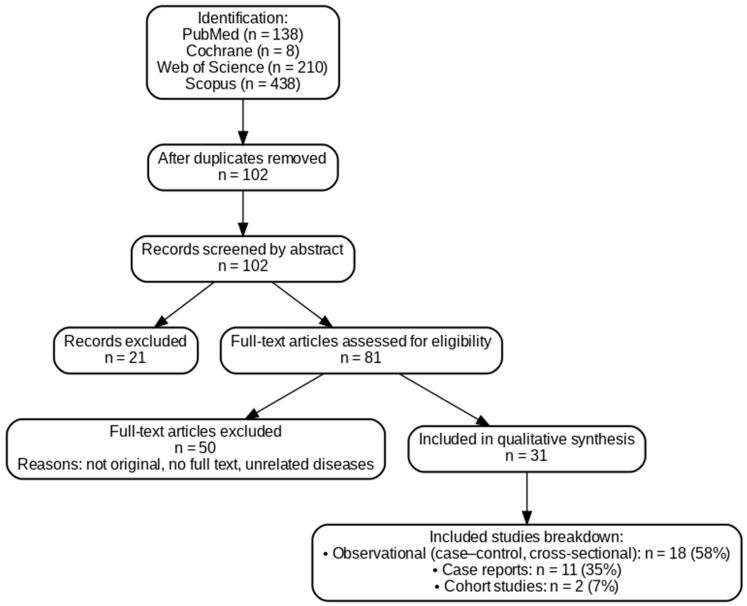
PRISMA-like flow diagram summarizing the study selection process for articles on ocular manifestations in celiac disease.

A comprehensive literature search was performed across four electronic databases. The PubMed database identified 138 records, the Cochrane database yielded 8 trials, the Web of Science search identified 210 records, and the Scopus search yielded 438 records. Following the removal of duplicates, 102 papers were selected for abstract screening; 21 were excluded based on keywords present in their abstracts, leaving 81 full-text papers to be evaluated for eligibility. Based on the exclusion criteria, 50 articles were excluded because they were not original studies, full text was not available, or they addressed unrelated diseases. Finally, 31 papers were included in the qualitative analysis. The overall selection process is summarized in the PRISMA-like flow diagram (Figure 1).

### 3.2. Characteristic of Inculded Studies

Altogether, 31 studies investigating ocular involvement in celiac disease were included in the qualitative synthesis. Observational studies, including case–control and cross-sectional designs, constituted the main source of evidence (n = 18; 58%), providing the most systematic data on ocular changes associated with celiac disease. Case reports accounted for 11 publications (35%), illustrating individual clinical observations and rare manifestations. Only two large cohort studies (7%) contributed high-level epidemiological evidence on the association between celiac disease and ocular outcomes. A detailed overview of the included studies covering study objectives, methodology, patient characteristics, key findings, and level of evidence is presented in Table 2.

### 3.3. Summary of Findings from Included Studies

Overall, the studies highlighted several consistent patterns. Evidence from a large cohort indicates a higher incidence of cataract and uveitis in patients with CD [26,51].

In the large Swedish cohort study by Mollazadegan et al., uveitis was identified using ICD registries without restriction to subtype. Chart review revealed that most documented cases represented unilateral acute anterior uveitis, consistent with common autoimmune-associated uveitic patterns. In this study, epidemiological data show a modest but significant increased risk of uveitis in patients with CD (HR of 1.32; 95% CI 1.10 to 1.58). It remains increased even 5 years after CD diagnosis (HR 1.31; 95% CI 1.04 to 1.64) [26]. Several case reports highlight that uveitis may present before or concurrently with gastrointestinal symptoms and resolve after adherence to GFD [27,28]. A recent retrospective case–control study reinforced this association, showing a higher prevalence of CD among patients with uveitis compared to the general population—OR of 5.77 (95% CI 1.4118 to 23.4737, *p* = 0.049) [29].

Moreover, CD is associated with an increased risk of developing cataracts [47,51,52]. This is evidenced by a study involving 28,000 individuals with CD, where the risk of cataract was 397/100,000 person-years, higher than the population risk of 86/100,000 person-years [51]. In a retrospective review of 272,873 ophthalmology records, Martins et al. identified cataract as one of the most frequent ocular diagnoses among patients with CD (12%), second only to dry eye disease (32%) [47]. Furthermore, a case report describing bilateral total cataract as the initial clinical presentation of previously undiagnosed CD highlights the possibility of lens involvement even in younger individuals [52]. Complementing these clinical observations, a study by Ozates et al. noted an increase in maximum optical density in children with CD (*p* = 0.028) [53].

A substantial number of studies focused on ocular surface alterations in patients with celiac disease. The most consistent findings included a reduced tear break-up time (TBUT) and decreased Schirmer test values, indicating tear film instability and aqueous deficiency. In pediatric cohorts, TBUT was significantly shorter compared with healthy controls—10.8 ± 3.8 s vs. 12.1 ± 1.7 s (*p* = 0.046) [31] and 10.86 ± 3.51 s vs. 15.25 ± 2.49 s (*p* < 0.001) [46]. Similar reductions were observed in adults—8.62 ± 2.74 s vs. 11.51 ± 2.31 s (*p* < 0.001) [32] and 10.1 ± 3.5 s vs. 12.3 ± 2 s (*p* < 0.05) [30].

Schirmer test results followed the same trend, with lower tear secretion reported in both children (17.9 ± 9.1 mm vs. 21.6 ± 4.1 mm; *p* = 0.038 [31]; 14.07 ± 5.14 mm vs. 20.20 ± 3.93 mm; *p* < 0.001 [46]) and adults (11.33 ± 2.83 mm vs. 14.29 ± 2.91 mm; *p* < 0.001 [32]; 8.2 ± 2.3 mm vs. 9.9 ± 1.7 mm; *p* < 0.05 [30]). Moreover, Hodžić et al. reported that consumption of foods containing trace amounts of gluten negatively affected tear film parameters, potentially predisposing to dry eye disease (DED) [33].

Structural abnormalities of the ocular surface have also been reported. In a case–control study, Uzel et al. demonstrated that adults with CD exhibited squamous metaplasia and goblet cell loss on impression cytology, with significantly higher mean cytology grades than controls (*p* = 0.001) [30].

Moreover, corneal nerve alterations have been explored. In children, a corneal confocal microscopy study found no significant differences in corneal nerve fiber density or length between CD patients and controls. However, there was a trend toward increased nerve tortuosity and reduced inferior whorl length, suggesting subtle subclinical changes [35].

Anterior segment alterations were also reported. Pediatric cohorts showed reduced anterior chamber depth (3.5 ± 0.2 vs. 3.7 ± 0.2; *p* < 0.001) and volume (170.8 ± 25.5 vs. 190.7 ± 27.4; *p* < 0.001) [31], whereas in adults, anterior chamber volume was increased 3.46 ± 0.31 vs. 3.31 ± 0.25; *p* = 0.037) and positively correlated with anti-gliadin IgA antibody levels (r = 0.369, *p* = 0.027) [32]. However, not all findings are consistent. A larger observational study reported no significant differences in anterior segment parameters between CD patients and healthy controls [34].

Several studies have assessed retinal and choroidal morphology in patients with CD using OCT and OCTA. In pediatric cohorts, Karatepe Hashas et al. reported a significant reduction in average RNFL thickness (102.8 ± 8.2 vs. 108.9 ± 10.1; *p* < 0.001) [31]. In Vatansever et al.’s study, RNFL was significantly thinner in the patient group compared to the control group (109.15 ± 9.94 vs. 100.11 ± 13.85; *p* < 0,001). Significant correlations were observed between cup/disk ratio and vitamin B12 (r = −0.0450, *p* < 0.001) and triglyceride (r = 0.310, *p* = 0.004) levels, as well as between RNFL thickness and ferritin (r = 0.540, *p* < 0.0001) levels [46]. In contrast, Isik et al. found no significant differences in overall RNFL or macular thickness [36].

Among adults, Hazar et al. identified significantly thinner superior RNFL (126.59 ± 14.19 µm vs. 134.18 ± 19.50 µm; *p* = 0.017) and thicker nasal RNFL (82.51 ± 8.68 vs. 78.57 ± 12.28; *p* = 0.007). The tissue transglutaminase 2 IgA antibody and superior RNFL thickness were negatively correlated (r = −0.394; *p* = 0.012) [32].

In pediatric cohorts, Dereci et al. demonstrated that all layers of the choroid in subfoveal, nasal, and temporal regions were significantly thinner in children with CD compared with healthy controls (*p* < 0.001 for all comparisons) [20]. Similarly, Doğan et al. emphasized that longer disease duration (>60 months), nonadherence to a GFD, and persistent antibody positivity were associated with more pronounced choroidal thinning [37]. In contrast to these observations, Bilgin et al. found no significant differences in choroidal, GCC, RNFL, or foveal thickness among pediatric CD patients, regardless of adherence to a gluten-free diet [19].

In contrast, studies conducted in adults have reported an opposite trend, with choroidal thickening rather than thinning. Bolukbasi et al. found significantly greater mean choroidal thickness in adult CD patients compared with controls (*p* < 0.001), with 84.2% of eyes demonstrating uncomplicated pachychoroid and one eye showing pachychoroid pigment epitheliopathy [38]. Consistent findings were reported by Bernardo et al., who observed increased subfoveal choroidal, luminal, and stromal areas (*p* < 0.001 for all) but no significant difference in the choroidal vascularity index [39].

OCTA-based studies further revealed microvascular alterations in CD. Several reports described reduced superficial and deep capillary plexus densities and altered peripapillary vessel density, with some correlations observed between vascular parameters, disease duration, and nutritional status [18,43]. Gumus et al. reported higher central vascular density (VD) values in both the superficial and deep capillary plexuses (*p* = 0.006 and *p* = 0.001, respectively), whereas temporal and nasal choriocapillaris densities were significantly lower in CD patients (*p* < 0.05 for both) [18]. Similarly, Erdem et al. showed that optic nerve head vessel density, radial peripapillary capillary VD, and deep capillary plexus VD were lower in CD compared with controls [43]. However, Isik et al. found no significant OCTA abnormalities in children with CD [36].

Finally, several rare ocular manifestations were identified, including orbital myositis [44], conjunctival tumor regressing under a GFD [45], central retinal vein occlusions [40,41,42], xerophthalmic fundus, and Bitot’s spots reported in case studies [48,49,50].

## 4. Discussion

In recent years, an increasing number of studies have explored the impact of immune-mediated diseases, including CD, on ocular health [47,54,55,56]. These investigations suggest that CD may affect multiple ocular structures. Nevertheless, emerging evidence indicates that several interrelated pathophysiological mechanisms may underlie ocular changes in CD. These mechanisms can be broadly categorized into three domains: immune-mediated pathways, nutritional and gut barrier–related factors, including micronutrient deficiencies and increased intestinal permeability, and vascular alterations, such as microangiopathy and prothrombotic states.

Understanding these mechanisms is clinically important, as ocular manifestations may occasionally represent the first or even the only sign of undiagnosed CD [28,40,41]. Early recognition and GFD adherence can help prevent persistent ocular symptoms and preserve visual function [20,27,28,37,44,45]. Despite increasing interest, the strength of current evidence remains limited, underscoring the need for rigorous, well-designed studies to validate these associations. The following sections provide a critical evaluation of the available literature, highlighting the biological plausibility of proposed mechanisms while also addressing methodological limitations and gaps that should be prioritized in future research.

### 4.1. Immunological Mechanisms

CD is an immune-mediated disorder triggered by gluten exposure in genetically predisposed individuals. It is characterized by a T-cell–driven response against deamidated gliadin. That leads to intestinal injury mediated by cytokines like IFN-γ, IL-15, IL-17, and with increased gut permeability allows systemic immune activation [1]. Extraintestinal manifestations, including ocular inflammation such as uveitis, may reflect this systemic immune dysregulation. Both CD and uveitis involve dysregulated T-helper cell subsets, especially Th1 and Th17, and overproduction of inflammatory cytokines [57].

These shared immune pathways, impaired barrier function, systemic T-cell activation, and cytokine-driven tissue injury suggest a “gut–eye axis.” Systemic inflammation associated with CD may facilitate the activation of autoreactive immune pathways within ocular tissues, thereby increasing the risk of uveitis in genetically or immunologically susceptible individuals. A similar gut–eye immune crosstalk has been described in inflammatory bowel disease (IBD), where disruption of the intestinal barrier, dysbiosis, and chronic immune activation contribute to ocular inflammation, particularly anterior uveitis [47,58,59]. This parallel strengthens the hypothesis that CD-associated ocular manifestations may not be incidental, but rather part of a broader spectrum of immune-mediated extraintestinal involvement.

Data from the literature indicate a significantly higher prevalence of uveitis among patients with celiac disease, as well as an increased frequency of celiac disease among patients with uveitis [26,29,47]. Uveitis may represent an isolated extraintestinal manifestation of CD, may precede the onset of gastrointestinal symptoms, or may occur concurrently with them. However, most available evidence originates from individual case reports [27,28], limiting the ability to draw firm conclusions. At present, there is insufficient evidence to support routine ophthalmologic screening for uveitis in all newly diagnosed CD patients. However, a targeted eye examination may be warranted in individuals with ocular symptoms, coexisting autoimmune diseases, or in cases of idiopathic ocular inflammation where celiac disease has not been excluded.

Among the most consistent ophthalmic findings in CD are tear film instability and ocular surface alterations, suggesting an immune-mediated disturbance of the lacrimal functional unit. Several studies have demonstrated a significant reduction in TBUT and decreased Schirmer test values in both pediatric and adult CD cohorts, indicating impaired tear film stability and aqueous deficiency despite the absence of overt ocular symptoms [30,31,32,46]. Persistent subclinical inflammation of the ocular surface, even in patients adhering to a gluten-free diet, underscores the need for ophthalmologic monitoring, particularly in those with symptoms such as dryness, burning, or fluctuating vision.

Notably, this pattern parallels ocular surface changes observed in autoimmune conditions such as Sjögren’s syndrome, which is also reported to co-occur more frequently in individuals with CD. Both conditions share features of mucosal immune dysregulation, chronic inflammation, and epithelial damage, and their ocular phenotypes overlap, particularly dry eye symptoms, goblet cell loss, and tear film instability [60,61]. Overall, these data suggest that tear film alterations in celiac disease are likely immune-mediated, reflecting both local inflammatory activity and systemic autoimmunity rather than being solely secondary to nutritional deficiency.

Several case–control and observational studies have investigated the anterior segment anatomy in CD [31,32,34]. From a clinical perspective, reduced chamber depth and volume are recognized risk factors for primary angle-closure glaucoma, yet direct data linking CD to this ocular outcome remain lacking [62]. Overall, while the available studies support an immunological contribution to ocular surface and anterior segment changes in CD, the strength of evidence is limited by small sample sizes and heterogeneity in patient populations. Larger, longitudinal studies are required to establish whether these alterations represent clinically relevant consequences of systemic immune dysregulation in CD.

Beyond anterior segment alterations, several studies have suggested structural changes in the RNFL in patients with CD [31,32,36,46]. These findings raise the possibility of subclinical neuroaxonal injury. Proposed mechanisms include chronic systemic inflammation, immune-mediated microvascular compromise, or antibody cross-reactivity with retinal neuronal antigens [31,32,36].

Similarly, observational studies have reported changes in choroidal thickness in both subfoveal and peripapillary regions, often detected in asymptomatic patients [20,37,38,39]. These structural findings may reflect the dual impact of immune activation and vascular dysregulation. Pro-inflammatory cytokines (IL-6, TNF-α, IL-17) and circulating immune complexes can promote endothelial dysfunction, leading to altered choroidal perfusion, increased vascular permeability, and secondary changes in retinal and choroidal thickness [17]. The observed variability—thinning in children and thickening in adults—may represent different stages of the same immunopathological process, with early microvascular loss progressing to chronic vascular remodeling.

Taken together, reports of RNFL thinning and choroidal alterations point toward the eye as a potential window into systemic immune dysregulation in CD. However, existing studies remain exploratory, with modest sample sizes and limited reproducibility. Larger, longitudinal investigations are needed to determine whether these ocular alterations represent true disease biomarkers or incidental findings. However, discrepancies across studies likely reflect methodological differences (e.g., OCT device and segmentation protocols), small sample sizes, and heterogeneity in disease duration or dietary adherence.

At the other end of the spectrum, rare neuro-ophthalmological presentations such as occipital cortical calcifications have been reported in association with CD. A case report of bilateral occipital calcification linked to longstanding CD and visual field loss potentially stemming from gliadin-triggered T-cell infiltration and blood–brain barrier disruption [63]. In broader neurology literature, cerebral calcifications in CD have also been discussed within the celiac disease–epilepsy–cerebral calcifications (CEC) syndrome context, where immune-mediated endothelial injury or folate malabsorption are implicated [64,65]. Although these examples are anecdotal and infrequent, they underscore that immune-mediated central nervous system injury in CD might manifest with visual consequences beyond typical eye pathology.

### 4.2. Nutritional Mechanisms and Gut Barrier Dysfunction

Nutritional deficiencies and gut barrier dysfunction, which remain common even in treated patients, represent another potential pathway linking CD to ocular involvement. Studies in individuals with micronutrient deficiencies suggest possible effects on ocular surface integrity, retinal metabolism, and neural function.

Among these, vitamin A deficiency has long been recognized as a cause of ocular surface disease, ranging from xerophthalmia and Bitot’s spots to more severe retinal changes [21,48,66,67]. While these manifestations are well documented in populations with malnutrition, reports directly linking CD to xerophthalmic findings are rare and largely anecdotal, limited to single case descriptions [48,49,50]. Caution is warranted when interpreting these findings, as serum vitamin A levels were not measured in the majority of studies evaluating ocular outcomes in CD. The evidence base remains limited to isolated case descriptions rather than controlled investigations.

More recently, attention has shifted to vitamin D, a micronutrient frequently deficient in CD. Low vitamin D levels have been associated with a broad spectrum of ocular disorders, including DED, cataract, myopia, age-related macular degeneration, glaucoma, diabetic retinopathy, thyroid eye disease, and uveitis [22,23,68]. Small observational and case–control studies have reported associations between low 25(OH)D serum concentration and structural alterations, such as increased subfoveal choroidal thickness, reduced choroidal vascularity index, and retinal thinning [69,70,71,72,73]. Taken together, these observations suggest that vitamin D plays an important role in ocular surface health, immune regulation, and vascular homeostasis. However, there is a notable lack of direct evidence linking vitamin D deficiency to specific ophthalmic complications in patients with CD, underscoring the need for targeted studies in this population.

Iron deficiency, commonly resulting from impaired duodenal absorption in CD, has been proposed to influence retinal structure and function via hypoxic stress and impaired mitochondrial activity. Evidence from systematic reviews and small OCT/OCTA-based studies suggests decreased RNFL thickness and alterations in retinal vessel density in individuals with iron deficiency anemia [25,46]. However, these findings are derived from heterogeneous populations and remain largely indirect. Importantly, no dedicated studies have evaluated these associations specifically in patients with celiac disease, and therefore, the relevance of iron deficiency–related ocular changes within this population remains uncertain.

Folate and vitamin B12 deficiencies, which are common in untreated CD and may persist despite adherence to a GFD, have been linked to cataract formation, optic neuropathy, RNFL thinning, and reduced retinal vascular density [24,46,51,74,75,76]. A key metabolic consequence of these deficiencies is impaired methylation and elevated circulating homocysteine. Hyperhomocysteinemia has been reported in both untreated and diet-treated CD and is biologically relevant due to its prothrombotic, prooxidative, and endothelial-toxic properties, which may further contribute to retinal and choroidal microvascular injury [14,77,78]. Although several case–control studies support these associations, their interpretation is limited by small sample sizes and heterogeneous patient populations, in which nutritional and autoimmune etiologies often coexist. This makes it difficult to determine the specific contribution of CD to the observed ocular changes. The main findings are summarized in Table 3, which provides an overview of ocular changes associated with nutritional imbalances, their possible pathomechanisms, and relevant references.

Other micronutrient deficiencies—such as zinc, copper, calcium, and magnesium—may also play a role in ocular pathology through mechanisms including oxidative stress, impaired collagen synthesis, and disrupted neurotransmission. These alterations have the potential to accelerate lens opacification or compromise retinal neuronal function [14,52,53]. However, the available evidence in this field is particularly limited, largely anecdotal, and often derived from heterogeneous gastrointestinal disease populations.

### 4.3. Vascular Mechanisms

Several studies have suggested that patients with CD may be at increased risk of cardiovascular complications, including cardiomyopathy, thromboembolic events, ischemic heart disease, stroke, and arrhythmias [79]. Similar mechanisms may extend to the eye, where microvascular alterations are increasingly recognized as a potential contributor to ocular pathology. Beyond immune-mediated endothelial dysfunction, metabolic factors, such as hyperhomocysteinemia, may promote a prothrombotic state and vascular injury, further compromising ocular perfusion and microcirculation. Compounding these effects, increased intestinal permeability and dysbiosis in CD may facilitate systemic spillover of inflammatory mediators (e.g., IL-6, TNF-α), further destabilizing vascular regulation and tissue oxygenation [77,78]. Supporting this hypothesis, case reports and small cohort studies have described retinal vein occlusion as an initial manifestation of previously undiagnosed CD, implicating both nutritional dysregulation and immune-mediated vascular injury in disease pathogenesis [40,41,42,78].

OCTA-based studies have also reported microvascular changes [18,36,43]. These observations support the hypothesis of CD-related microangiopathy. However, the evidence remains preliminary. Methodological variability, small sample sizes, and the predominantly cross-sectional design of available studies substantially limit causal inference. Larger, prospective longitudinal investigations are required to clarify whether these vascular alterations represent reversible changes with dietary treatment or permanent structural damage.

### 4.4. Limitations of the Study

This review has several limitations. Although the literature search was extended to multiple databases, the possibility of missing relevant studies cannot be completely excluded, especially non-English publications. Many available studies on ocular manifestations of CD are based on small cross-sectional cohorts or isolated case reports, often without adjustment for confounding variables such as age, refractive status, or comorbidities. Heterogeneity in imaging methodologies, patient selection, and outcome measures further limits comparability across studies. Moreover, many of the included studies were conducted in patients already on GFD for varying periods of time, which may have influenced ocular findings. Adherence to GFD is rarely quantified and is inherently difficult to assess objectively, adding further uncertainty to the interpretation of results. In addition, the analysis was descriptive rather than a formal meta-analysis; therefore, the findings should be interpreted with caution. Finally, the review was limited to selected ophthalmic parameters and covered studies available up to April 2025, so more recent evidence may not have been captured.

### 4.5. Future Research Directions

Future research should prioritize large, prospective, and longitudinal studies, including patients without treatment. Particular attention should be given to evaluating circulating levels of vitamins, trace elements, and immune parameters to better disentangle the relative contribution of malabsorption, systemic inflammation, and vascular changes to ocular changes in CD. Integration of nutritional, immunological, and imaging biomarkers may help clarify mechanisms and enable the identification of reliable disease biomarkers. Collaborative multicenter efforts could overcome sample size limitations and enhance reproducibility.

## 5. Conclusions and Clinical Implications

**Figure 2:**
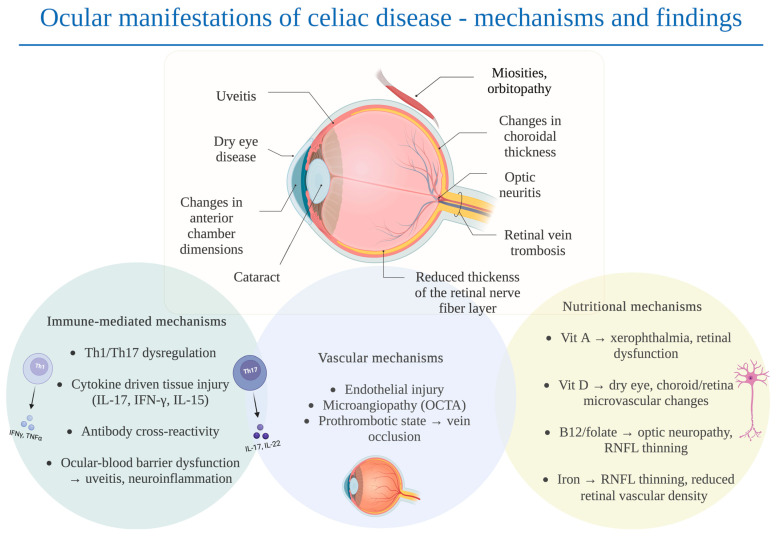
Scheme of ocular manifestations of celiac disease with possible pathomechanisms. Created in BioRender. Senterkiewicz, M. (2025).

In summary, CD is a systemic disorder that can affect multiple organs, including the eye. Current evidence suggests that immune dysregulation, nutritional deficiencies, the presence of circulating autoantibodies, and microvascular alterations may all contribute to ocular involvement in CD. Large population-based cohort studies indicate an increased risk of uveitis and cataract, while smaller cross-sectional and case–control imaging studies have reported tear film instability, ocular surface abnormalities, reduced anterior chamber depth and volume, alterations in subfoveal and peripapillary choroidal thickness, sectoral thinning of the peripapillary RNFL, and microvascular changes. More rare complications, such as myositis, orbitopathy, optic neuritis, and retinal vein thrombosis, have so far been described only in isolated case reports or very small series. A schematic summary of the ocular manifestations of celiac disease and the potential underlying pathomechanisms is presented in Figure 2, providing a visual framework to support the conclusions of this review.

### Practical Recommendations for Clinicians

In patients with confirmed CD, referral for ophthalmic evaluation should be considered in cases of eye pain, vision loss, ocular surface discomfort, or unexplained visual disturbances. Periodic eye examinations may be particularly relevant for patients with delayed diagnosis, poor dietary adherence, or concurrent autoimmune conditions.

In patients without known CD, the diagnosis should be considered in cases of otherwise unexplained symptoms like uveitis (especially recurrent or bilateral), retinal vascular occlusion in young individuals, optic neuritis of unclear etiology, or ocular findings accompanied by systemic symptoms such as chronic diarrhea, weight loss, or unexplained iron deficiency anemia.

For both groups, early detection and management of ocular complications may prevent bothersome ocular symptoms, improve visual function, and protect against abnormalities in ocular development. Furthermore, recognition of ocular manifestations could prompt earlier diagnosis of CD.

## Figures and Tables

**Table 1:** Clinical subtypes of celiac disease according to current nomenclature [1,9,10].

Subtype of Celiac Disease	Definition	Typical Clinical Presentation
Classical	Intestinal mucosal damage with positive celiac-specific serology and symptoms resulting from malabsorption	Diarrhea, abdominal distension, poor growth in children, anemia, nutrient deficiencies, failure to thrive, delayed puberty; may include extraintestinal manifestations
Non-classical	Intestinal mucosal damage with positive serology but without overt malabsorption symptoms	Extraintestinal manifestations, such as iron deficiency anemia, osteoporosis, liver enzyme elevation, neurologic symptoms, infertility, dental enamel defects, and gastrointestinal symptoms, may be mild or absent
Subclinical	Positive serology and intestinal damage without clinical symptoms	Often discovered through screening (family members, associated autoimmune diseases, type 1 diabetes, Down syndrome); symptoms below the threshold of clinical detection, often having clinical (enamel defects…) and/or laboratory signs (iron deficiency anemia, liver function abnormalities)
Asymptomatic	Positive serology with intestinal injury in patients with no clinical or laboratory signs or symptoms	Often discovered through screening; no response to gluten withdrawal
Potential	Positive celiac-specific antibodies and genetic susceptibility, but a normal intestinal biopsy	May be symptomatic or asymptomatic; some progress to overt celiac disease, especially if persistently exposed to gluten

**Table 2:** Summary of published studies on ocular manifestations in celiac disease with level of evidence.

Authors and Year	Aim of Studies	Types of Studies Included	Number of Eyes/Patients Included	Summary of Results	Level of Evidence	Ref. No.
Gumus et al., 2022	Retinal and choroidal vasculature in newly diagnosed CD	Cross-sectional case–control OCTA study (DRI-OCT Triton, Topcon)	44 right eyes of patients newly diagnosed with CD and 44 healthy patients	↑ central section of the superficial and deep vascular plexuses; reduced temporal and nasal vessel density for choriocapillaris; thinner choroid in CD, more evident in females	Moderate	[18]
Bilgin & Sahin, 2023	OCT findings in pediatric CD with adherence to GFD	Cross-sectional pediatric OCT study (Optovue RTVue OCT)	68 eyes of 34 patients (14 patients who adhere to GFD, 20 who do not adhere to GFD)	Adhering to a GFD does not make any difference in choroidal, GCC, RNFL, and foveal thicknesses in pediatric CD patients	Moderate	[19]
Dereci et al., 2021	OCT findings in pediatric CD	Cross-sectional case–control study SD-OCT (Spectralis, Heidelberg)	43 CD patients and 48 healthy children	After 1 year of GFD in CD patients: thinning of the subfoveal, nasal, and temporal areas of the choroid	Moderate	[20]
Mollazadegan et al., 2012	Risk of uveitis in CD	Nationwide cohort study	>28,000 CD patients vs. controls	CD associated with ↑ the risk of uveitis, even 5 years after CD diagnosis	High	[26]
Krifa et al., 2010	Case: uveitis in a child with CD and diabetes mellitus type 1	Case report	1 patient	Uveitis resolved with GFD	Low	[27]
Klack et al., 2011	Case: uveitis in an adult with CD	Case report	1 patient	Uveitis remission after GFD	Low	[28]
Milstein et al., 2023	Risk of CD in uveitis patients	Multicenter observational study	112 patients with non-infectious uveitis	Increased risk of CD in uveitis patients	Moderate	[29]
Uzel et al., 2017	Ocular surface parameters in adult CD	Cross-sectional case–control study	56 eyes of 28 CD patients and 58 eyes of 29 healthy adults	Tear-film functions and conjunctival surface epithelial morphology altered in CD patients	Moderate	[30]
Karatepe Hashas et al., 2017	Ocular involvement in children with CD	Cross-sectional case–control pediatric study SD-OCT (Spectralis, Heidelberg)	31 CD children and 34 children in the control group	↓ in RNFL thickness; anterior chamber shallowing; qualitative and quantitative reduction in tears	Moderate	[31]
Hazar et al., 2021	Ocular parameters in adult CD patients	Cross-sectional case–control study (Optovue RTVue OCT)	58 eyes of 31 CD patients and 50 eyes of 50 controls	In CD group: lower tear film parameters, changed segmental RNFL thickness, and larger anterior chamber depth	Moderate	[32]
Hodžić et al., 2024	Dry eye in CD	Observational study (research for a randomized clinical trial)	100 CD patients	Subjective and objective measures of dry eye were lower in CD patients who consumed food that may contain gluten	Moderate	[33]
De Bernardo et al., 2022	Anterior segment and corneal parameters in CD	Observational case–control study (IOL Master, Pentacam HR)	70 CD patients and 70 healthy controls	No major corneal and anterior segment changes between the two groups	Moderate	[34]
Gad et al., 2020	Corneal nerves in pediatric CD	Cross-sectional case–control study (confocal microscopy)	20 CD pediatric patients and 20 healthy controls	Minimal evidence of neuropathy in children with CD	Moderate	[35]
Isik et al., 2022	Retinal vasculature of the retina and choroid in children with CD	Cross-sectional case–control OCTA study	60 CD pediatric patients and 71 healthy controls	No statistically significant differences in vessel density, FAZ, and choroidal thickness	Moderate	[36]
Doğan et al., 2020	Choroidal thickness and adherence to GFD	Cross-sectional case–control OCT study (SD-OCT, Retinascan RS-3000, Nidek)	42 CD patients and 42 healthy controls	Longer duration of GFD was associated with adherence difficulty and thinner choroidal thickness	Moderate	[37]
Bolukbasi et al., 2019	Choroidal thickness and pachychoroid in CD	Cross-sectional case–control SD-OCT study (Spectralis, Heidelberg)	70 CD patients and 70 healthy controls	↑ choroidal thickness in CD patients	Moderate	[38]
De Bernardo et al., 2021	Choroidal structure in CD	Cross-sectional case–control EDI-OCT study (Spectralis, Heidelberg)	74 CD patients and 67 healthy patients	↑ choroidal thickness in CD patients (increased vascular and stromal components)	Moderate	[39]
Malhi et al., 2018	CRVO in CD	Case reports	2 patients	CRVO led to CD diagnosis	Low	[40]
Zoubeidi et al., 2016	CRVO in CD	Case report	1 patient	Non-ischemic CRVO as the first sign of CD	Low	[41]
Lee & Pulido, 2005	CRVO in CD	Case report	1 patient	Non-ischemic CRVO in the CD patient	Low	[42]
Erdem et al., 2022	Retinal microvascular changes in CD	Cross-sectional case–control study (Optovue AngioVue OCT)	30 CD patients and 30 controls	Reduced vessel density of the optic nerve head and radial peripapillary capillary;	Moderate	[43]
Cerman et al., 2014	Orbital myositis in CD	Case report	1 patient	Myositis improved with GFD and immunosuppression	Low	[44]
Tuncer et al., 2010	Regression of conjunctival tumor with GFD in CD	Case report	1 pediatric patient	Tumor regressed after GFD—presumed diagnosis of conjunctival Kaposi sarcoma	Low	[45]
Vatansever et al., 2022	Lab parameters and ocular findings in pediatric CD	Cross-sectional case–control study (SD-OCT, Retinascan RS-3000, Nidek)	100 eyes of 50 pediatric CD patients and 110 eyes of 55 healthy controls	↓ Schirmer test and TBUT results in the CD group, positively correlated with Vit D; ↓ macular and RNFL thickness in the CD group	Moderate	[46]
Martins dos Santos et al., 2022	Eye disorders in CD/IBD (data warehouse)	Cross-sectional retrospective data study	272,873 patients with eye disease were evaluated between 2003 and 2019	↑ prevalence of anterior uveitis, cataract, dry eye, diabetic retinopathy, pathological myopia, and AMD in CD	Moderate	[47]
Sharma et al., 2014	Bitot’s spots and CD	Case report	1 child	Bitot’s spots → Vit A deficiency → CD	Low	[48]
Witherspoon & Callanan, 2008	Xerophthalmic fundus in CD	Case report	1 patient	Vit A deficiency fundus improved with Vit A + GFD	Low	[49]
Pereira et al., 2024	Vit A deficiency retinopathy in CD + liver fibrosis	Case report	1 patient	Severe retinopathy in a CD patient from Vit A deficiency	Low	[50]
Mollazadegan et al., 2011	Risk of cataract in CD	Population cohort study	28,756 CD patients	↑ risk of cataract (HR ~1.3)	High	[51]
Raina et al., 2011	Bilateral cataract as the first sign of CD	Case report	1 patient	Bilateral, total, subluxated cataract in a young CD patient	Low	[52]
Ozates et al., 2019	Corneal and lens density in pediatric CD	Cross-sectional case–control study (Pentacam HR)	50 CD patients and 51 healthy controls	↑ maximum lens density in patients with CD; ↑ peripheral corneal density in female patients with CD	Moderate	[53]

Abbreviations: CD—Celiac disease; OCTA—Optical coherence tomography angiography; DRI-OCT—Deep range imaging optical coherence tomography; GFD—Gluten-free diet; GCC—Ganglion cell complex; RNFL—Retinal nerve fiber layer; SD-OCT—Spectral-domain optical coherence tomography; FAZ—Foveal avascular zone; EDI-OCT—Enhanced depth imaging optical coherence tomography; CRVO—Central retinal vein occlusion; TBUT—Tear break-up time; Vit D—Vitamin D; IBD—Inflammatory bowel disease; AMD—Age-related macular degeneration; Vit A—Vitamin A. Level of evidence: high—population-based cohort studies; moderate—cross-sectional studies; low—case reports or case series.

**Table 3:** Ocular changes associated with nutritional imbalances, possible pathomechanisms, and references.

Nutrient Deficiency	Ocular Manifestations	Proposed Mechanism	Strength of Evidence
Vitamin A	Xerophthalmia Bitot’s spots Nyctalopia Conjunctival keratinization Keratomalacia	Loss of goblet cells, Squamous metaplasia Rod dysfunction	Systematic review [21] Narrative review [66] Observational study [67] Case reports, anecdotal observations [48,49,50]
Vitamin D	Dry eye disease Cataract Myopia Age-related macular degeneration Glaucoma Diabetic retinopathy, Thyroid eye disease Uveitis Increased choroidal thickness RNFL thinning	Immunomodulation (Th1/Th17 regulation), Calcium homeostasis, Endothelial function	Systematic reviews [22,23,68]; Cross-sectional and small case–control OCT studies [69,70,71,72,73]
Iron	RNFL thinning Smaller foveal avascular zone (OCTA) Reduced capillary plexus density (OCTA)	Disrupted neuronal metabolism Ischemia	Systematic review and meta-analysis [25], Case–control and OCT study [46]
Folate and vitamin B12	Cataract Optic neuropathy RNFL thinning Reduced retinal vascular density (OCTA) Increased risk of central retinal vein occlusion	Neurodegeneration Retinal microcirculation impairment	Case–control and OCT studies [24,46,74,75,76]; Epidemiology (cataract risk) [51]

Abbreviations: RNFL—Retinal nerve fiber layer; OCT—Optical coherence tomography; OCTA—Optical coherence tomography angiography.

## Abbreviations

The following abbreviations are used in this manuscript (in order of appearance in the text):

CD	Celiac disease
GFD	Gluten-free diet
IBD	Inflammatory bowel disease
OCT	Optical coherence tomography
OCTA	Optical coherence tomography angiography
RNFL	Retinal nerve fiber layer
TBUT	Tear break-up time
SD-OCT	Spectral-domain optical coherence tomography
EDI-OCT	Enhanced depth imaging optical coherence tomography
GCC	Ganglion cell complex
FAZ	Foveal avascular zone
CRVO	Central retinal vein occlusion
VD	Vascular density

## References

[1] Lebwohl B., Sanders D.S., Green P.H.R. (2018). Coeliac Disease. Lancet.

[2] Singh P., Arora A., Strand T.A., Leffler D.A., Catassi C., Green P.H., Kelly C.P., Ahuja V., Makharia G.K. (2018). Global Prevalence of Celiac Disease: Systematic Review and Meta-Analysis. Clin. Gastroenterol. Hepatol..

[3] Repo M., Koskimaa S., Paavola S., Kurppa K. (2025). Serological Testing for Celiac Disease in Children. Expert. Rev. Gastroenterol. Hepatol..

[4] Jericho H., Sansotta N., Guandalini S. (2017). Extraintestinal Manifestations of Celiac Disease: Effectiveness of the Gluten-Free Diet. J. Pediatr. Gastroenterol. Nutr..

[5] Szaflarska-Popławska A., Dolińska A., Kuśmierek M. (2022). Nutritional Imbalances in Polish Children with Coeliac Disease on a Strict Gluten-Free Diet. Nutrients.

[6] Di Nardo G., Villa M.P., Conti L., Ranucci G., Pacchiarotti C., Principessa L., Raucci U., Parisi P. (2019). Nutritional Deficiencies in Children with Celiac Disease Resulting from a Gluten-Free Diet: A Systematic Review. Nutrients.

[7] Wessels M., Dolinsek J., Castillejo G., Donat E., Riznik P., Roca M., Valitutti F., Veenvliet A., Mearin M.L. (2022). Follow-up Practices for Children and Adolescents with Celiac Disease: Results of an International Survey. Eur. J. Pediatr..

[8] Lebwohl B., Green P.H.R., Söderling J., Roelstraete B., Ludvigsson J.F. (2020). Association Between Celiac Disease and Mortality Risk in a Swedish Population. JAMA.

[9] Caio G., Volta U., Sapone A., Leffler D.A., De Giorgio R., Catassi C., Fasano A. (2019). Celiac Disease: A Comprehensive Current Review. BMC Med..

[10] Ludvigsson J.F., Leffler D.A., Bai J.C., Biagi F., Fasano A., Green P.H.R., Hadjivassiliou M., Kaukinen K., Kelly C.P., Leonard J.N. (2013). The Oslo Definitions for Coeliac Disease and Related Terms. Gut.

[11] Jericho H., Guandalini S. (2018). Extra-Intestinal Manifestation of Celiac Disease in Children. Nutrients.

[12] Dominguez Castro P., Harkin G., Hussey M., Christopher B., Kiat C., Liong Chin J., Trimble V., McNamara D., MacMathuna P., Egan B. (2017). Changes in Presentation of Celiac Disease in Ireland from the 1960s to 2015. Clin. Gastroenterol. Hepatol..

[13] Volta U., Caio G., Stanghellini V., De Giorgio R. (2014). The Changing Clinical Profile of Celiac Disease: A 15-Year Experience (1998–2012) in an Italian Referral Center. BMC Gastroenterol..

[14] Fousekis F.S., Katsanos A., Katsanos K.H., Christodoulou D.K. (2020). Ocular Manifestations in Celiac Disease: An Overview. Int. Ophthalmol..

[15] dos Santos Martins T.G., de Azevedo Costa A.L.F., Oyamada M.K., Schor P., Sipahi A.M. (2016). Ophthalmologic Manifestations of Celiac Disease. Int. J. Ophthalmol..

[16] Vujosevic S., Parra M.M., Hartnett M.E., O’Toole L., Nuzzi A., Limoli C., Villani E., Nucci P. (2022). Optical Coherence Tomography as Retinal Imaging Biomarker of Neuroinflammation/Neurodegeneration in Systemic Disorders in Adults and Children. Eye.

[17] Steiner M., Esteban-Ortega M.d.M., Muñoz-Fernández S. (2019). Choroidal and Retinal Thickness in Systemic Autoimmune and Inflammatory Diseases: A Review. Surv. Ophthalmol..

[18] Gumus M., Eker S., Karakucuk Y., Ergani A.C., Emiroglu H.H. (2022). Retinal and Choroidal Vascular Changes in Newly Diagnosed Celiac Disease: An Optical Coherence Tomography Angiography Study. Indian. J. Ophthalmol..

[19] Bilgin B., Sahin Y. (2023). Impact of Adherence to Gluten-Free Diet in Paediatric Celiac Patients on Optical Coherence Tomography Findings: Ocular Imaging Based Study. Photodiagnosis Photodyn. Ther..

[20] Dereci S., Asik A., Direkci I., Karadag A.S., Hizli S. (2021). Evaluation of Eye Involvement in Paediatric Celiac Disease Patients. Int. J. Clin. Pract..

[21] Castro-Pachón S., Perilla-Soto S., Ruiz-Sarmiento K., Niño-García J.A., Sánchez-Rosso M.J., Ordóñez-Caro M.C., Camacho-Páez D.S., García-Lozada D. (2025). Prevalence of Ocular Manifestations of Vitamin A Deficiency in Children: A Systematic Review. Arch. Soc. Esp. Oftalmol..

[22] Liu J., Dong Y., Wang Y. (2020). Vitamin D Deficiency Is Associated with Dry Eye Syndrome: A Systematic Review and Meta-Analysis. Acta Ophthalmol..

[23] Jue Z., Xu Z., Yuen V.L., Chan O.D.S., Yam J.C. (2025). Association between Vitamin D Level and Cataract: A Systematic Review and Meta-Analysis. Graefes Arch. Clin. Exp. Ophthalmol..

[24] Ayyildiz T., Dulkadiroglu R., Yilmaz M., Polat O.A., Gunes A. (2021). Evaluation of Macular, Retinal Nerve Fiber Layer and Choroidal Thickness by Optical Coherence Tomography in Children and Adolescents with Vitamin B12 Deficiency. Int. Ophthalmol..

[25] Ghasemi M., Ghasemi A., Khorasani S., Zare S., Sazgar A.K., Nikkhah H. (2024). Characteristics of Optical Coherence Tomography in Patients with Iron Deficiency Anemia: A Systematic Review and Meta-Analysis. BMC Ophthalmol..

[26] Mollazadegan K., Kugelberg M., Tallstedt L., Ludvigsson J.F. (2012). Increased Risk of Uveitis in Coeliac Disease: A Nationwide Cohort Study. Br. J. Ophthalmol..

[27] Krifa F., Knani L., Sakly W., Ghedira I., Essoussi A.S., Boukadida J., Ben Hadj Hamida F. (2010). Uveitis Responding on Gluten Free Diet in a Girl with Celiac Disease and Diabetes Mellitus Type 1. Gastroenterol. Clin. Biol..

[28] Klack K., Pereira R.M.R., De Carvalho J.F. (2011). Uveitis in Celiac Disease with an Excellent Response to Gluten-Free Diet: Third Case Described. Rheumatol. Int..

[29] Milstein Y., Haiimov E., Slae M., Davidovics Z., Millman P., Birimberg-Schwartz L., Benson A., Wilschanski M., Amer R. (2023). Increased Risk of Celiac Disease in Patients with Uveitis. Ocul. Immunol. Inflamm..

[30] Uzel M.M., Citirik M., Kekilli M., Cicek P. (2017). Local Ocular Surface Parameters in Patients with Systemic Celiac Disease. Eye.

[31] Karatepe Hashas A.S., Altunel O., Sevınc E., Duru N., Alabay B., Torun Y.A. (2017). The Eyes of Children with Celiac Disease. J. AAPOS.

[32] Hazar L., Oyur G., Atay K. (2021). Evaluation of Ocular Parameters in Adult Patients with Celiac Disease. Curr. Eye Res..

[33] Hodžić N., Banjari I., Mušanović Z., Nadarević-Vodenčarević A., Pilavdžić A., Kurtćehajić A. (2024). Accidental Exposure to Gluten Is Linked with More Severe Dry Eye Disease in Celiac Disease Patients on a Gluten-Free Diet. New Armen. Med. J..

[34] De Bernardo M., Vitiello L., Gagliardi M., Capasso L., Rosa N., Ciacci C. (2022). Ocular Anterior Segment and Corneal Parameters Evaluation in Celiac Disease. Sci. Rep..

[35] Gad H., Saraswathi S., Al-Jarrah B., Petropoulos I.N., Ponirakis G., Khan A., Singh P., Khodor S.A., Elawad M., Almasri W. (2020). Corneal Confocal Microscopy Demonstrates Minimal Evidence of Distal Neuropathy in Children with Celiac Disease. PLoS ONE.

[36] Isik I., Yaprak L., Yaprak A., Akbulut U. (2022). Optical Coherence Tomography Angiography Findings of Retinal Vascular Structures in Children with Celiac Disease. J. AAPOS.

[37] Doğan G., Şen S., Çavdar E., Mayalı H., Cengiz Özyurt B., Kurt E., Kasırga E. (2020). Should We Worry about the Eyes of Celiac Patients?. Eur. J. Ophthalmol..

[38] Bolukbasi S., Erden B., Cakir A., Bayat A.H., Elcioglu M.N., Yurttaser Ocak S., Gokden Y., Adas M., Asik Z.N. (2019). Pachychoroid Pigment Epitheliopathy and Choroidal Thickness Changes in Coeliac Disease. J. Ophthalmol..

[39] De Bernardo M., Vitiello L., Battipaglia M., Mascolo F., Iovino C., Capasso L., Ciacci C., Rosa N. (2021). Choroidal Structural Evaluation in Celiac Disease. Sci. Rep..

[40] Malhi R., Dhami A., Malhi N., Soni A., Dhami G. (2018). Central Retinal Vein Occlusion Revealing Celiac Disease: The First Report of Two Cases from India. Indian. J. Ophthalmol..

[41] Zoubeidi H., Ben Salem T., Ben Ghorbel I., Houman M.H. (2016). Central Retinal Vein Occlusion Revealing Coeliac Disease. Eur. J. Case Rep. Intern. Med..

[42] Lee E.S., Pulido J.S. (2005). Nonischemic Central Retinal Vein Occlusion Associated with Celiac Disease. Mayo Clin. Proc..

[43] Erdem S., Ucmak F., Karahan M., Ava S., Dursun M.E., Dursun B., Hazar L., Yolaçan R., Keklikci U. (2022). Evaluation of Retinal Microvascular Perfusion Changes in Patients with Celiac Disease. Ocul. Immunol. Inflamm..

[44] Cerman E., Esen F., Eraslan M., Kazokoglu H. (2014). Orbital Myositis Associated with Celiac Disease. Int. Ophthalmol..

[45] Tuncer S., Yeniad B., Peksayar G. (2010). Regression of Conjunctival Tumor during Dietary Treatment of Celiac Disease. Indian. J. Ophthalmol..

[46] Vatansever M., Dursun Ö., Tezol Ö., Dinç E., Vatansever E., Sari A., Usta Y. (2022). Effects of Laboratory Parameters on Tear Tests and Optical Coherence Tomography Findings in Pediatric Celiac Disease. Duzce Med. J..

[47] Martins T.G.d.S., Miranda Sipahi A., dos Santos F.M., Schor P., Anschütz A., Mendes L.G.A., Silva R. (2022). Eye Disorders in Patients with Celiac Disease and Inflammatory Bowel Disease: A Study Using Clinical Data Warehouse. Eur. J. Ophthalmol..

[48] Sharma A., Aggarwal S., Sharma V. (2014). Bitot’s Spots: Look at the Gut. Int. J. Prev. Med..

[49] Witherspoon S.R., Callanan D. (2008). Celiac Disease Presenting as a Xerophthalmic Fundus. Retina.

[50] Pereira A., Wright T., Weisbrod D., Ballios B.G. (2024). Vitamin A Deficiency Retinopathy in the Setting of Celiac Disease and Liver Fibrosis. Doc. Ophthalmol..

[51] Mollazadegan K., Kugelberg M., Lindblad B.E., Ludvigsson J.F. (2011). Increased Risk of Cataract among 28,000 Patients with Celiac Disease. Am. J. Epidemiol..

[52] Raina U.K., Goel N., Sud R., Thakar M., Ghosh B. (2011). Bilateral Total Cataract as the Presenting Feature of Celiac Disease. Int. Ophthalmol..

[53] Ozates S., Doguizi S., Hosnut F.O., Sahin G., Sekeroglu M.A., Yilmazbas P. (2019). Assessment of Corneal and Lens Density in Children with Celiac Disease. J. Pediatr. Ophthalmol. Strabismus.

[54] Glover K., Mishra D., Singh T.R.R. (2021). Epidemiology of Ocular Manifestations in Autoimmune Disease. Front. Immunol..

[55] Santonicola A., Wieser H., Gizzi C., Soldaini C., Ciacci C. (2024). Associations between Celiac Disease, Extra-Gastrointestinal Manifestations, and Gluten-Free Diet: A Narrative Overview. Nutrients.

[56] Moshirfar M., Melanson D.G., Pandya S., Moin K.A., Talbot C.L., Hoopes P.C. (2024). Implications of Celiac Disease in Prospective Corneal Refractive Surgery Patients: A Narrative Review. Cureus.

[57] Meng T., Nie L., Wang Y. (2025). Role of CD4+ T Cell-Derived Cytokines in the Pathogenesis of Uveitis. Clin. Exp. Med..

[58] Campagnoli L.I.M., Varesi A., Barbieri A., Marchesi N., Pascale A. (2023). Targeting the Gut-Eye Axis: An Emerging Strategy to Face Ocular Diseases. Int. J. Mol. Sci..

[59] Pytrus W., Akutko K., Pytrus T., Turno-Kręcicka A. (2022). A Review of Ophthalmic Complications in Inflammatory Bowel Diseases. J. Clin. Med..

[60] Ayar K., Tunç R., Pekel H., Esen H.H., Küçük A., Çifçi S., Ataseven H., Özdemir M. (2020). Prevalence of Sicca Symptoms and Sjögren’s Syndrome in Coeliac Patients and Healthy Controls. Scand. J. Rheumatol..

[61] Beas R., Altamirano-Farfan E., Izquierdo-Veraza D., Norwood D.A., Riva-Moscoso A., Godoy A., Montalvan-Sanchez E.E., Ramirez M., Guifarro D.A., Kitchin E. (2024). Prevalence of Celiac Disease in Systemic Lupus Erythematosus, Sjögren Syndrome and Systemic Sclerosis: A Systematic Review and Meta-Analysis. Dig. Liver Dis..

[62] Zhou S., Pardeshi A.A., Burkemper B., Apolo G., Cho A., Jiang X., Torres M., McKean-Cowdin R., Varma R., Xu B.Y. (2023). Refractive Error and Anterior Chamber Depth as Risk Factors in Primary Angle Closure Disease: The Chinese American Eye Study. J. Glaucoma.

[63] Millington R.S., James-Galton M., Barbur J.L., Plant G.T., Bridge H. (2015). Severe, Persistent Visual Impairment Associated with Occipital Calcification and Coeliac Disease. J. Neurol..

[64] Arroyo H.A., De Rosa S., Ruggieri V., de Dávila M.T.G., Fejerman N. (2002). Epilepsy, Occipital Calcifications, and Oligosymptomatic Celiac Disease in Childhood. J. Child. Neurol..

[65] Pfaender M., D’Souza W.J., Trost N., Litewka L., Paine M., Cook M. (2004). Visual Disturbances Representing Occipital Lobe Epilepsy in Patients with Cerebral Calcifications and Coeliac Disease: A Case Series. J. Neurol. Neurosurg. Psychiatry.

[66] Smith J., Steinemann T.L. (2000). Vitamin A Deficiency and the Eye. Int. Ophthalmol. Clin..

[67] Chiu M., Dillon A., Watson S. (2016). Vitamin A Deficiency and Xerophthalmia in Children of a Developed Country. J. Paediatr. Child. Health.

[68] Chan H.N., Zhang X.J., Ling X.T., Bui C.H.T., Wang Y.M., Ip P., Chu W.K., Chen L.J., Tham C.C., Yam J.C. (2022). Vitamin D and Ocular Diseases: A Systematic Review. Int. J. Mol. Sci..

[69] Vural E., Hazar L., Çağlayan M., Şeker Ö., Çelebi A.R.C. (2021). Peripapillary Choroidal Thickness in Patients with Vitamin D Deficiency. Eur. J. Ophthalmol..

[70] Gürbostan Soysal G., Berhuni M., Özer Özcan Z., Tıskaoğlu N.S., Kaçmaz Z. (2023). Decreased Choroidal Vascularity Index and Subfoveal Choroidal Thickness in Vitamin D Insufficiency. Photodiagnosis Photodyn. Ther..

[71] Öncül H., Alakus M.F., Çağlayan M., Öncül F.Y., Dag U., Arac E. (2020). Changes in Choroidal Thickness after Vitamin D Supplementation in Patients with Vitamin D Deficiency. Can. J. Ophthalmol..

[72] Aydemir E., Ilhan C., Aksoy Aydemir G., Bayat A.H., Bolu S., Asik A. (2022). Evaluation of Retinal Structure in Pediatric Subjects with Vitamin D Deficiency. Am. J. Ophthalmol..

[73] Icel E., Ucak T., Ugurlu A., Erdol H. (2022). Changes in Optical Coherence Tomography Angiography in Patients with Vitamin D Deficiency. Eur. J. Ophthalmol..

[74] Özkasap S., Türkyilmaz K., Dereci S., Öner V., Calapoǧlu T., Cüre M.C., Durmuş M. (2013). Assessment of Peripapillary Retinal Nerve Fiber Layer Thickness in Children with Vitamin B12 Deficiency. Childs Nerv. Syst..

[75] Icel E., Ucak T. (2021). The Effects of Vitamin B12 Deficiency on Retina and Optic Disk Vascular Density. Int. Ophthalmol..

[76] Koca S., Bozkurt E., Dogan M., Yavasoglu F., Erogul Ö., Bulut A.K. (2023). Effects of B12 Deficiency Anemia on Radial Peripapillary and Macular Vessel Density: An Optical Coherence Tomography Angiography (OCTA) Study. Klin. Monbl. Augenheilkd..

[77] Fousekis F.S., Beka E.T., Mitselos I.V., Milionis H., Christodoulou D.K. (2021). Thromboembolic Complications and Cardiovascular Events Associated with Celiac Disease. Ir. J. Med. Sci..

[78] Pantic N., Pantic I., Jevtic D., Mogulla V., Oluic S., Durdevic M., Nordin T., Jecmenica M., Milovanovic T., Gavrancic T. (2022). Celiac Disease and Thrombotic Events: Systematic Review of Published Cases. Nutrients.

[79] Ciaccio E.J., Lewis S.K., Biviano A.B., Iyer V., Garan H., Green P.H. (2017). Cardiovascular Involvement in Celiac Disease. World J. Cardiol..

